# The New York Institute of Stomatology

**Published:** 1900-05

**Authors:** 

**Affiliations:** The New York Institute of Stomatology


### Reports of Society Proceedings.

### THE NEW YORK INSTITUTE OF STOMATOLOGY.

A REGULAR meeting of the Institute was held on Tuesday evening, February 6, 1900, at the office of Dr. E. A. Bogue, No. 63 West Forty-eighth Street, New York, the President, Dr. E. A. Bogue, in the chair.

The minutes of the previous meeting were read and approved.

### COMMUNICATIONS ON THEORY AND PRACTICE.

*Dr. W. St. George Elliott.*—I would like to ask if any of the members of the Institute have any knowledge of the use of the ethylate of sodium. Some years ago my attention was called to it, and I have used it ever since. It is very corrosive, and injures the stoppers of the bottles so that it is often impossible to get them out. It is useful in cases where it is necessary to remove a portion of gum lying in a cavity: a piece of cotton saturated with the ethylate of sodium and applied to the gum removes it effectually in a gelatinous mass, and generally without pain. It is also a very excellent medicament for cleansing root-canals.

*Dr. Geo. S. Allan.*—Where is it obtained?

*Dr. Elliott.*—It was originally imported from England, but now, in the form of a powder, it can be obtained from any druggist.

*Dr. J. Morgan Howe.*—Is it preferable to caustic potash?

*Dr. Elliott.*—I have never used it in the same place. I rather fancy it has not at all the same action. When I was practising in London, a young lady came into my office with a small tumor on the upper lip, just over the left lateral incisor, and which was just about the size of a pea. I did not investigate it microscopically, but asked her to allow me to remove it with caustics. She objected, and consulted a surgeon. He pronounced it a very serious growth, and stated that she would have to undergo a painful operation, attended with the loss of one or more teeth and a portion of the process, and that even then there was a liability of recurrence. The next time she called at my office I again asked to be allowed to experiment with it. She consented, and I applied the ethylate of sodium. The tumor disappeared and has never returned.

*The President.*—If there is nothing further to be said under this head, we will proceed to the essayists of the evening. I am pleased to announce that we have with us this evening Dr. Horatio C. Meriam, of Salem, Mass., who will describe to us a new suspension crown. I take pleasure in introducing Dr. Meriam.

(For Dr. Meriam's paper, see page 293.)

DISCUSSION.

*Dr. F. Milton Smith.*—I would like to ask what gutta-percha Dr. Meriam finds to be most useful for his work; also the kind of gold he uses, and what the guage is?

*Dr. Meriam.*—I find that the most useful form of gutta-percha is a combination of oxide of zinc, six parts, and ordinary Sumatra hard white gutta-percha, one part, softening this as I use it with any of the essential oils. I frequently put a drop of oil of sassafras in a well in my porcelain slab, and dip my gutta-percha into it before introducing. I prefer gutta-percha for this work because the piece can be so easily removed. The gold I use is eighteen parts of pure gold, five parts of silver and one part of copper. This I have a refiner roll out for me to about a 30 gauge, and when more strength is required I use two or more thicknesses.

*Dr. Smith.*—I would like to ask Dr. Meriam if he makes these open face bands to fit as tightly as he can to the tooth which they are to cover, or does he leave a space for the gutta-percha?

*Dr. Meriam.*—That depends upon the condition. For an extension crown it is necessary for the band to fit tightly, but in making a bridge with a suspension crown this is not so important, and the band may be a little large and filled with gutta-percha, so that the appliance can be easily removed.

*The President.*—Dr. Frederic L. Bogue is unable to be with us to-night. He has, however, sent his paper, which Dr. Davenport has kindly consented to read.

*Dr. S. E. Davenport.*—Dr. Frederic L. Bogue has prepared the little paper which I have the pleasure of presenting to you. I

am sorry to say that he has been ill for the past two or three days, and regrets very much not being able to be with us to-night. The subject of his paper is "A Modified Wire Clasp."

### A MODIFIED WIRE CLASP.

BY DR. FREDERIC L. BOGUE.

Mr. President and Gentlemen,—Before presenting the little modification, which at best would only be applicable to a very limited number of cases, I think it would not be amiss to call attention to the ordinary wire clasp, which, so far as I can learn, is not in general use.

I believe our fellow member Professor J. P. Michaels, of Paris, was the first to suggest its use abroad. At about the same time, Dr. W. S. Elliott, of Sag Harbor, patented and advertised it in this country. Although working independently, the forms of clasp devised are almost identical. The guage of the wire used in these clasps is from nineteen to twenty-one, depending upon the relative degree of rigidity or elasticity required. The wire furnished by Aderer Bros. is very satisfactory, inasmuch as it does not lose its elasticity when annealed, as most of the other makes do.

Some of the advantages of these clasps are: lightness combined with strength; great elasticity; are easily bent to fit teeth of any shape, especially convex teeth, the lower wire fitting under the tuberosity, the upper wire above it; minimum contact with the surface of the tooth, which is an advantage because a tooth bearing a clasp is injured not only by mechanical abrasion, but by the chemical decomposition of the *débris* held in contact with the tooth by the clasp.

The modification I wish to present this evening was designed for a case in which the back tooth tipped forward, forming a space which was largest at the gum margin. The idea was to make a fixture easily inserted and removed, and yet one that would fit the teeth when in position.

The clasp for the back tooth was made and attached to the plate, as though no undercut existed. The standard for the forward clasp was soldered the thickness of the clasp wire back of the front edge of the plate. This is done in order that the plate might extend as far forward as possible without having the clasp wire interfere. The upper end of the standard was bent forward almost at a right angle, in such a way that when the upper wire was soldered to the front of the bent end of the standard, the wire would be in contact with the tooth. The object of bending the standard in that way was to prevent the fixture from being driven forward during the act of mastication. The forward clasp, which was shaped like a hairpin, was fitted to the tooth. The upper wire was soldered to the standard; the lower wire, being left free and fitting the neck of the tooth, was brought far enough around to be accessible from the front. When it is desired to remove the fixture, the lower wire is pressed back against the standard and the plate lifted up from the front end.

*Dr. F. J. McLaren.*—I have used these clasps very extensively, and in certain cases find them almost invaluable. The fact that a firm attachment to the tooth can be combined with a great amount of resiliency is in itself a marked advantage. They are very useful in cases where there is a single tooth, as a molar or a canine, remaining, and in those where it is desirable to combine retention by clasp with suction. We all know how the ordinary clasp will interfere with the suction of a plate, but with the wire clasp there is such mobility that this interference is overcome. There is one slight objection to these clasps, and that is the fact that the ends cannot be cut off. I overcome this difficulty by soldering pieces of gold on the ends instead of making an all-wire clasp.

*Dr. J. Bond Littig.*—Are these simply wire loops?

*Dr. McLaren.*—They are double loops attached at one point. They were at first only made with one loop. Dr. Elliott, who patented them, made them in some twelve or fifteen patterns. I use the straight clasp wire, beginning at one end and contouring it along until I have it the desired shape, then bringing it back in the same way to where I started.

*Dr. L. C. Leroy.*—I find these wire clasps of the greatest advantage in attaching such appliances as removable bridges, holding the appliance firm and giving least amount of contact with the tooth. The only teeth to which they cannot be attached are the canines. They are especially adapted to bell-shaped teeth.

*The President.*—I am pleased to say, in reply to Dr. Leroy, that I put in a gold plate last November for a gentleman eighty-six years old. Only the six front teeth remained on the upper jaw. I clasped the canines, using the wire clasp, and the plate was very successful.

*Dr. Leroy.*—The impression I meant to convey was that the canines were ill adapted for these clasps as compared with the other teeth. Of course, it is not impossible to clasp them in this manner.

*Dr. Charles A. Meeker.*—I would like to say that when Dr. Elliott first introduced these clasps I bought some, and have used all varieties ever since, more especially in rubber work. I use for this work German silver wire, which I obtain from Patterson Brothers, Park Row, New York. I find the German silver wire adheres more firmly to the rubber and is more easily contoured to the tooth, altogether giving better results than the gold wire.

*The President.*—In Dr. Bogue's modification I would call attention to the free end of the wire. This end is either melted down into the shape of a knob, or else a drop of solder is placed at the end of the wire and melted, leaving the free end of the wire with a small smooth knob.

*Dr. J. F. P. Hodgson.*—I would like to ask if any one can use these clasps, or if there is a patent on them.

*The President.*—I wrote to Dr. Elliott when the patent was first published, telling him that I had then been using these clasps for a number of years, and warning him that there was no use trying to uphold a patent if he had one. I can thank Dr. Michaels, of Paris, for the idea, as he gave it to me some years before Dr. Elliott brought it out.

*Dr. Elliott.*—It has been said of a certain warm country, that it is paved with good intentions. It was my intention to make several drawings of the cases I am to bring before you to-night, but unfortunately I was prevented from doing so. The three cases came to me in the one week.

A gentleman had broken his central incisor by an accident. I found the parts very much inflamed. I gave him the choice of a bridge, a plate, or an implanted tooth. He chose the latter. With difficulty I found a tooth of the proper shape, and after cutting it off, fitted to the root a backed porcelain crown of the proper shade and shape. The only thing new was in the way I made the splint, which was swaged from silver, covering the cutting edges of the teeth; but instead of making holes for the ligatures in the splint, it was split at intervals corresponding with the spaces of the teeth.

After being properly fitted, it was thickly covered with oxyphosphate and placed in position. It was very readily applied, and answered the purpose very well indeed.

Another case was that of an upper left bicuspid, which I had prepared for a Logan crown by cementing a cylinder into the root. The operation seemed successful, but inside of four days the patient came back with the cylinder out, and I then found the root was split. After again applying the cylinder, I made a hoop of platinized gold which fitted very closely, and this I drove on with a hammer, and the crown was again inserted. Since then I have had no further trouble.

Another case was that of erosion involving the labial surfaces of the two upper centrals. It was the worst case of erosion that I have ever seen. There was, of course, great difficulty in obtaining proper anchorage. I did this to a certain extent by cutting shallow grooves all about the edges of the teeth, and then, by using the ordinary process of a platinum matrix, I baked a porcelain facing, which on being placed in position very much improved the condition.

The last case which I wish to mention was that of a split molar. It was split nearly in the centre. I drilled a hole through it and passed through a German silver bar, on one end of which was a head, which I countersunk into the tooth. On the other end was a thread, to which a nut was fitted. This was put in with cement, and after it had been in position for a few days the nut was ground off, the tooth being retained by the cement. Generally a shell crown is preferable, however.

*Dr. Hodgson.*—I am reminded of a very mortifying experience of my own in this direction fully twenty-five years ago. I had bolted together with infinite care and pains the split shells of a large molar, and built into it a strong filling, enclosing the bolt. In a few days the whole sides had split away in the other direction. It became my decided opinion that a tooth which could so split, being generally an old pulpless one, does not often possess sufficient toughness of structure to admit of its large sides being held together reliably by a tiny bolt-head. I always thereafter employed a narrow gold or platinum band, according to whether gold or amalgam were to be used for filling, perfectly fitted and set with oxychloride or gutta-percha and having horizontal arms reaching from it into the large cavity to be enclosed in the after filling.

This was in the old days before the gold crowns. I should not feel it to be justifiable to use this method at present, when we are able to get almost perfect adjustment and lasting strength for these back teeth with properly adapted gold crowns.

*The President.*—I should like to ask Dr. Hodgson whether he considers the friability of the tooth of which he speaks due in any degree to the loss of the pulp.

*Dr. Hodgson.*—Possibly not so entirely on the fact that the pulp was dead as upon the additional fact that this death of the pulp has been followed by a decay of the dentine extending nearly to the enamel, which, as we all know, is a friable combination very easily fractured.

*Dr. Meriam.*—A tooth very frequently met with in this split condition is the first bicuspid, and it is sometimes a very difficult one to deal with. One of this kind I treated by means of a strong platinum-iridium staple set in the roots with phosphate cement. The tooth gave service for many years. A split molar was also treated in this manner by placing pins in the canals. The tooth was crowned and has lasted for fifteen years.

*Dr. Charles O. Kimball.*—I have nothing particularly new to offer, but there are one or two cases which I should like to mention. The first case is that of an upper second molar which had decayed and had been patched and filled so many times that it had finally broken off and the three roots stood apart. They were all in a loose and very bad condition. I first sterilized them and got them in good condition. I then made of platinum wire a three-pointed tack, each point corresponding to a canal. This was set into the roots with cement, and around this was built oxyphosphate cement.

It has been in now for six years and is still doing good service. The roots, which were so exceedingly loose, have become firm, and it has been a useful tooth ever since. I think now, were I to do the same operation, I should use amalgam instead of the cement; the result, however, has been very satisfactory. The other illustrates the advantage of sticking to a case. Four years ago a lady about fifty years of age had up to that time been wearing an artificial upper central incisor since she was a girl of seventeen. It was a pivot tooth set on a firm root. The cavity of the root a good many years ago had become enlarged, and I had had a tube set in it. While she was abroad she noticed that this root was offensive. On her return I found that the tooth had split, going a little to one side, and as the pivot tooth had been set with gutta-percha, there was quite a separation, over one-thirty-second of an inch. The split apparently, so far as I could judge, went up in an oblique direction, and the tube which was set in the root was still firm in the larger portion. I drew the pieces together with silk, taking two or three days to do it, and proceeding very gently, as it was very sensitive. I could not, however, approximate them entirely. The best I could do was to get them together at the lower end, and after trying for several days I had to content myself with getting the band around the end of the root. There was a great deal of irritation and discharge. I saw a friend of mine, who advised implantation, but I concluded to wait a little while before doing this. I gradually succeeded in sterilizing the tooth, and the discharge finally ceased. In making the crown for this tooth I had it so made that a little ridge of gum covered the band completely. My friend, who wanted to implant a tooth, said he thought it would not last over two years. It has gone for over three years, and the root is in better shape now than it was in the beginning.

*Dr. Howe.*—I have used a method of treating molar roots that have become separated, which has served me well for a number of years. After the root-canals have been treated and any existing abscess cured, in each root a strong stiff pin is set in cement, letting it stand up above the end of the root. These are then drawn together by means of a contractile thread, such as the grass-line. After which they are held in position by binding wire around the pins. Leaving this wire in position, I place a matrix around the roots at the gum margin and fill with amalgam, leaving the matrix on till the amalgam sets. A gold cap may then be adapted to make more perfect contour if desired. In that way I have restored very badly broken down molars, and in many cases the results have been most satisfactory. The appearance of some such amalgam crowns, and of amalgam fillings too, after ten to twenty years of service, I consider a refutation of the claims of amalgam-makers that there is no good amalgam but that made after the most modern ideas.

*Dr. Elliott.*—Regarding that last remark, I wish to state that recently I have been making a good many experiments with amalgams, particularly with the "Fellowship" and "Twentieth Century," and I find them no better than the old amalgams. I recall a case of a second lower molar, the roots of which had separated and were in an unhealthy condition. After sterilizing and getting in good condition, without attempting to draw the two together I put a crown on the root resting on the gum. The crown is now in perfect condition and has been in position for eighteen years.

*Dr. B. C. Nash.*—I recall one case which caused me a great deal of concern, as it occurred early in my practice,—that of a lower molar with a cavity on the buccal surface. The pulp was dead and the canals had been skilfully approached through this buccal opening. I carried on the treatment, filled the roots, and finally inserted in the buccal cavity a gold filling. Two days afterwards the patient returned with the filling out. On examination I found that the tooth had split directly across. I fitted a gold band around the tooth and filled it with amalgam. I have never seen the patient since, and received no pay for the operation. This experience taught me not to experiment with split teeth.

*Dr. Allan.*—I have a tooth here which I obtained years ago at the old Johnson depot. It is a split molar, and has a peculiar history. The man in charge related the history, offering the tooth for sale at five dollars. It seems that this tooth had caused the patient severe pain for several weeks, but the dentist could find absolutely nothing the matter with it. One day the fellow came in and said he was cured; that he was all right now. He said "the d—d thing exploded." How this happened I do not know, but apparently, according to this history, relief was obtained after the tooth split and the pressure was gotten rid of.

*Dr. Meriam.*—I have had a similar case of great pressure in a tooth. The case was a central incisor, which I was drilling into. On withdrawing the drill after passing it into the pulp chamber, it was followed by the entire contents of the canal.

*The President.*—One of the gentlemen in the course of the discussion spoke of wishing to implant a tooth and not finding one of the proper color. I had a similar experience two or three years ago, and finally was obliged to implant one which was not the exact shade. The shade of this tooth has changed, and last year when I saw it the color was exactly right. I should like to know of the gentlemen who have had experience in implanting teeth, if this is the usual case.

*Dr. Hodgson.*—I wish to call attention to the usefulness of touching the warmed gutta-percha on its way to the cavity with the oil of cajeput, on account of the increased stickiness of the gutta-percha so treated, such a filling actually cementing itself to the walls of the cavity. It can even be applied wet, and so is of real value in treating a patient ill in bed, etc. The experiment has been performed of sticking a piece of gutta-percha so softened to the inside of a tumbler filled with water, and after cooling it can only be removed with difficulty. It is useful in applying temporary stoppings where there is little or no retention. Also in those cavities which extend clear around a tooth and are so difficult to fill. In this latter case, however, and, indeed, wherever I need more lasting filling along with the cemented quality, I have devised a method which is far superior to the foregoing, in that the gutta-percha is not injured in the least. It is to flood the dried cavity with the oil of cajeput and absorb out the excess, leaving the walls damp; then packing into it the warmed gutta-percha, holding the filling if necessary with an instrument held in the left hand until the packing is completed.

Adjourned.

FRED. L. BOGUE, M.D., D.D.S.,
*Editor The New York Institute of Stomatology.*

